# Humulone Modulation of GABA_A_ Receptors and Its Role in Hops Sleep-Promoting Activity

**DOI:** 10.3389/fnins.2020.594708

**Published:** 2020-10-14

**Authors:** Ali Y. Benkherouf, Kim Eerola, Sanna L. Soini, Mikko Uusi-Oukari

**Affiliations:** Integrative Physiology and Pharmacology, Institute of Biomedicine, University of Turku, Turku, Finland

**Keywords:** GABAA receptors, humulone, ethanol, allosteric modulation, radioligand binding, electrophysiology, sleep

## Abstract

*Humulus lupulus* L. (hops) is a major constituent of beer. It exhibits neuroactive properties that make it useful as a sleeping aid. These effects are hypothesized to be mediated by an increase in GABA_A_ receptor function. In the quest to uncover the constituents responsible for the sedative and hypnotic properties of hops, recent evidence revealed that humulone, a prenylated phloroglucinol derivative comprising 35–70% of hops alpha acids, may act as a positive modulator of GABA_A_ receptors at low micromolar concentrations. This raises the question whether humulone plays a key role in hops pharmacological activity and potentially interacts with other modulators such as ethanol, bringing further enhancement in GABA_A_ receptor-mediated effects of beer. Here we assessed electrophysiologically the positive modulatory activity of humulone on recombinant GABA_A_ receptors expressed in HEK293 cells. We then examined humulone interactions with other active hops compounds and ethanol on GABA-induced displacement of [^3^H]EBOB binding to native GABA_A_ receptors in rat brain membranes. Using BALB/c mice, we assessed humulone’s hypnotic behavior with pentobarbital- and ethanol-induced sleep as well as sedation in spontaneous locomotion with open field test. We demonstrated for the first time that humulone potentiates GABA-induced currents in α1β3γ2 receptors. In radioligand binding to native GABA_A_ receptors, the inclusion of ethanol enhanced humulone modulation of GABA-induced displacement of [^3^H]EBOB binding in rat forebrain and cerebellum as it produced a leftward shift in [^3^H]EBOB displacement curves. Moreover, the additive modulatory effects between humulone, isoxanthohumol and 6-prenylnaringenin were evident and corresponded to the sum of [^3^H]EBOB displacement by each compound individually. In behavioral tests, humulone shortened sleep onset and increased the duration of sleep induced by pentobarbital and decreased the spontaneous locomotion in open field at 20 mg/kg (*i.p*.). Despite the absence of humulone effects on ethanol-induced sleep onset, sleep duration was increased dose-dependently down to 10 mg/kg (*i.p*.). Our findings confirmed humulone’s positive allosteric modulation of GABA_A_ receptor function and displayed its sedative and hypnotic behavior. Humulone modulation can be potentially enhanced by ethanol and hops modulators suggesting a probable enhancement in the intoxicating effects of ethanol in hops-enriched beer.

## Introduction

**FIGURE 1:**
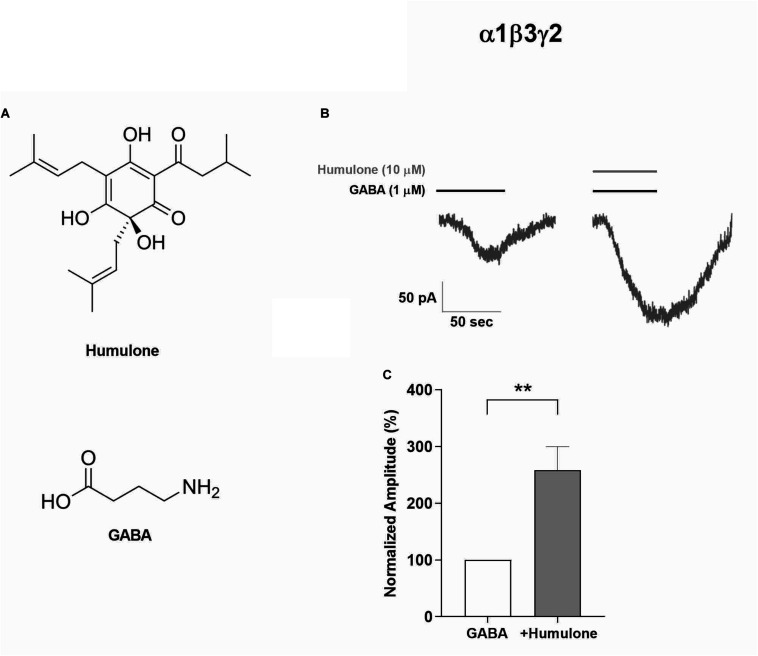
Enhancement of GABA-induced currents by humulone in recombinant α1β3γ2 GABA_A_ receptors expressed in HEK293 cells and voltage-clamped at −60 mV, pH 7.4. **(A)** Chemical structures of humulone and GABA. **(B)** Representative current traces of receptor activation upon 1 min application of a submaximal concentration of GABA (1 μM) in the presence or absence of humulone (10 μM). **(C)** Bar graph illustrating the peak current amplitude normalized to that induced by the presence of 1 μM GABA alone. Each vertical bar represents mean ± SEM, *n* = 8 cells recorded. **p < 0.01 for the significance of difference from GABA only application (paired *t*-test).

Hops, the resinous female flowers of the plant *Humulus lupulus* L., are widely used as a major ingredient for beer brewing. The lupulin glands of hops secrete yellow powder of prenylated phloroglucinol derivatives, known as alpha acids, which are essential for foam stability, bitterness and preservation of beer (Verzele and De Keukeleire, 1991; Van Cleemput et al., 2009). These alpha acids mainly consist of humulone (Figure 1A, 35–70% of total alpha acids), cohumulone (20–65%), and adhumulone (10–15%) (Neve, 1991; Verzele and De Keukeleire, 1991; Ntourtoglou et al., 2020). The therapeutic potential of alpha acids has been investigated for their wide range of bioactivity against bacteria, osteoporosis, angiogenesis, inflammation and cancer as comprehensively reviewed (Van Cleemput et al., 2009; Karabín et al., 2016). Furthermore, alpha acids were found to exhibit sedative and hypnotic properties (Zanoli et al., 2005; Schiller et al., 2006), indicating their major role in hops’ sleep-promoting activity previously reported in animal models (Bravo et al., 1974; Lee et al., 1993; Franco et al., 2012a, 2014) and humans (Vonderheid-Guth et al., 2000; Dimpfel and Suter, 2008; Franco et al., 2012b). This activity is attributed to the positive modulation of γ-aminobutyric acid type A (GABA_A_) receptor function demonstrated earlier with hops extracts (Aoshima et al., 2006; Sahin et al., 2016) and alpha acid fractions (Benkherouf et al., 2020).

GABA_A_ receptors, members of the Cys-loop superfamily of ligand-gated ion channels, are responsible for the fast-acting inhibitory synaptic transmission in the brain (Uusi-Oukari and Korpi, 2010; Sigel and Steinmann, 2012; Speigel et al., 2017). These heteropentameric protein complexes assemble from 8 subunit classes encoded by 19 distinct genes: α1-α6, β1-β3, γ1-γ3, δ, ε, π, θ, and ρ1-ρ3 (Korpi et al., 2002; Rudolph and Möhler, 2006; Whiting, 2006; Goetz et al., 2007). Upon receptor activation, the intrinsic chloride channel is opened leading to chloride ion influx, which hyperpolarizes the membrane potential resulting in neuron inhibition (Olsen and Sieghart, 2008). Hence, the modulation of GABA_A_ receptor function is an important mechanism to induce and maintain sedation, sleep and anesthesia as well as alcohol intoxication (Sieghart, 1995; Aguayo et al., 2002; Wallner et al., 2006a; Förstera et al., 2016).

In the quest to uncover the constituents responsible for the sedative and hypnotic properties of hops, we earlier isolated hops fractions with semipreparative liquid chromatography and revealed individual components that modulate [^3^H]EBOB binding to native GABA_A_ receptors with variable potency. The humulone fraction was found to display potent modulatory activity at low micromolar concentrations in rat forebrain membranes (IC_50_ = 3.2 ± 0.4 μM) with insensitivity to flumazenil antagonism and low potency on the benzodiazepine binding site (Benkherouf et al., 2020). This prompted further exploration of its role in hops enhancement of GABA_A_ receptor function. Given that humulone dietary intake in humans occurs mainly through hopped beer consumption, this raises the question whether humulone potentially interacts with other modulators such as ethanol and hops flavonoids, bringing further enhancement in GABA_A_ receptor-mediated effects of beer.

Here, we assessed the modulatory activity of humulone using electrophysiological measurements in recombinant GABA_A_ receptors expressed in HEK293 cells. We further examined humulone-ethanol enhancements with [^3^H]ethynylbicycloorthobenzoate (EBOB) binding assay in native and recombinant GABA_A_ receptors and tested humulone interactions with other bioactive hops modulators. We finally evaluated humulone’s hypnotic and sedative behavior using pentobarbital/ethanol-induced sleep and open field tests in BALB/cAnNRj mice.

## Materials and Methods

### Reagents

The radioligand [propyl-2,3-^3^H]ethynylbicycloorthobenzoate ([^3^H]EBOB, specific activity 48 Ci/mmol) was purchased from Perkin Elmer Life and Analytical Sciences (Boston, MA, United States). GABA and picrotoxin were obtained from Sigma Chemical Co. (St. Louis, MO, United States). Humulone was purchased from Specs (Zoetermeer, Netherlands), 6-prenylnaringenin and isoxanthohumol were purchased from PhytoLab (Vestenbergsgreuth, Germany), and sodium pentobarbital solution for injection (Mebunat Vet^®^, 60 mg/mL) was from Orion Corporation (Espoo, Finland). Ethanol was from Altia (Rajamäki, Finland).

### Animals

Native male Sprague-Dawley rats (11–13 weeks of age) used in binding assays and male BALB/cAnNRj mice (9–11 weeks of age) used in behavioral tests, were both purchased from the University of Turku Central Animal Laboratory (UTUCAL). The animals were housed in standard conditions (12 h light-dark cycle at 21 ± 1°C and humidity 65%) and they had access to standard rodent chow food and water *ad libitum*. Animal care and maintenance were according to the Finnish Act on Animal Experimentation (62/2006), European legislation (2010/63/EU), and OECD Principles of Good Laboratory Practice [ENV/MC/CHEM(98)17]. Rats were euthanized by decapitation; their fore/midbrain and cerebellum were dissected, frozen on dry ice, and stored at −70°C. All experimental procedures in this study were carried out under the approval of the Animal Experiment Board in Finland (license number: ESAVI/25715/2018).

### Recombinant GABA_A_ Receptor Expression in HEK293 Cells

Human embryonic kidney (HEK) 293 cells (Sigma- Aldrich, St Louis, MO, United States) were maintained in Dulbecco’s modified Eagle’s medium supplemented with 10% fetal bovine serum (Gibco, Gaithersburg, MD; United States), 50 U/mL penicillin and 50 μg/mL streptomycin (Sigma-Aldrich, St Louis, MO, United States) under standard growth conditions at 37°C, 95% humidity and 5% CO_2_. The cells were divided and plated on 150 mm culture dishes for binding assays and 12 mm coverslips in 24-well cell culture plates for electrophysiology 24 h before transfection.

The cells were transiently transfected with rat cDNAs encoding GABA_A_ receptor subunits (α1, L08490; α6, L08495; β3, X15468; γ2S, L08497; δ, L08496) in pRK5 plasmids under the control of CMV promoter (Uusi-Oukari et al., 2000). The plasmids were used in 1:1 and 1:1:1 ratio for transfections containing α1β3γ2S, α6β3, α6β3γ2S, and α6β3δ receptor subtypes. For radioligand binding, CaPO_4_ precipitation method was used essentially as described earlier (Lüddens and Korpi, 1997). The integration of γ2 subunit in the assembled receptors was verified using [^3^H]Ro 15–4513 binding in principle as described in Uusi-Oukari and Korpi (1990) (Supplementary Figure S1). For electrophysiology, K4^®^ transfection system (Biontex, München, Germany) was used according to the manufacturer’s protocol to increase expression efficiency and cell viability. The plasmid pWPI containing EGFP marker (Addgene, 12254) was co-transfected to identify the green fluorescent transfected cells in electrophysiological recordings. The medium was changed 24 h after transfection and all experiments were performed 48 h after transfection.

### HEK293 Cell Electrophysiology

Whole-cell electrophysiology was performed for moderately EGFP positive single cells. The cells were routinely clamped at –60 mV using an Axopatch 200B amplifier (Molecular Devices, Sunnyvale, CA, United States) and the Strathclyde Electrophysiology Software Package WinWCP (University of Strathclyde, United Kingdom). Patch pipettes (3–5 MΩ) were pulled from the thin-wall borosilicate glass tubing (1.5/1.12 mm; OD/ID) (WPI, Sarasota, FL, United States) on a P-87 Flaming Brown micropipette puller (Sutter Instrument Company, Rafael, CA, United States), and filled with an internal solution containing the following (in mM) 150 CsCl, 2 MgCl_2_, 1.1 EGTA, 2 Mg-ATP, 10 HEPES, pH 7.4 adjusted with 1 mM CsOH.

The cells were continuously perfused with external HEK-Krebs solution containing (in mM): 140 NaCl, 4.7 KCl, 1.2 MgCl_2_, 2.52 CaCl_2_, 11 glucose, and 5 HEPES, pH 7.4 adjusted with 1 mM NaOH. GABA (1 μM) and humulone (10 μM) were prepared as stock solutions and diluted to the desired concentrations with the external solution on the day of the experiment and bath-applied for 1 min. The external solution and drug solutions were driven by gravity at the flow rate of 5 mL/min and the current responses were recorded at room temperature (20–22°C). Peak currents were measured directly from the baseline to the peak response and normalized to that induced by GABA alone for each recorded cell.

### [^3^H]EBOB Binding Assay

Rat fore/midbrain and cerebellar membranes were prepared according to Squires and Saederup (2000) modified protocol as described previously (Benkherouf et al., 2019). Frozen membranes were thawed, washed once by centrifugation at 20,000 g for 10 min at +4°C in the assay buffer (50 mM Tris-HCl, 120 mM NaCl, pH 7.4), and finally resuspended in the same buffer. The protein concentrations of brain membranes were determined with the Bio-Rad Coomassie blue dye-based protein assay kit (Hercules, CA, United States) as per the manufacturer’s protocol. Transfected HEK293 cells were harvested 48 h after transfection using a detaching buffer (10 mM Tris-HCl, 0.15 M NaCl, 2 mM EDTA) and centrifuged at 20,000 g for 10 min at +4 °C. The resulting pellets were suspended in the assay buffer for binding assays.

Triplicate samples of rat fore/midbrain and cerebellar membranes were incubated at room temperature with shaking for 120 min in assay buffer with [^3^H]EBOB (1 nM) and different concentrations of GABA (50 nM–20 μM) in the presence of humulone (1 μM) or ethanol (30 mM) or both in a total volume of 400 μL. In humulone concentration series with recombinant α6β3, α6β3γ2S, and α6β3δ receptors, HEK293 cell membranes were incubated in the same above conditions for 120 min with [^3^H]EBOB (1 nM) and different concentrations of humulone (30 nM–30 μM) in the presence or absence of ethanol (30 mM). Since 1 nM [^3^H]EBOB contains up to 0.39 mM ethanol, EtOH-free [^3^H]EBOB was prepared by dry evaporating [^3^H]EBOB, solubilizing it in 5 μL DMSO and further in the appropriate assay buffer volume. Picrotoxin (100 μM) was used to determine the non-specific binding in all assays.

Membrane samples were filtered through Whatman GF/B glass fiber filters (Whatman International Ltd., Maidstone, United Kingdom) using a Brandel Cell Harvester (model M-24, Gaithersburg, MD, United States). The filters were rinsed three times with 5 mL of ice-cold 10 mM Tris-HCl, pH 7.4 buffer. Air-dried filters were immersed in 3 mL of Hidex AquaLight Beta scintillation liquid and radioactivity was determined in Hidex 600 SL liquid scintillation counter (Hidex, Turku, Finland).

### Pentobarbital- and Ethanol-Induced Sleep Tests

To evaluate the hypnotic activity of humulone, male BALB/cAnNRj mice (9–11 weeks of age) were pretreated intraperitoneally (*i.p.*) with either 10 or 20 mg/kg humulone (solubilized in 40% propylene glycol/5% Tween 80) or vehicle (40% propylene glycol/5% Tween 80) before sleep induction with either pentobarbital sodium or ethanol. Pentobarbital sodium (35 mg/kg, *i.p.*) or ethanol (3.5 g/kg, *i.p.*) diluted in 0.9% physiological saline was administered 45 min after humulone/vehicle treatment. As an index of drug-induced CNS inhibition, sleep latency (time between pentobarbital administration and loss of righting reflex) and duration of sleep (time between loss and recovery of righting reflex) were measured with a chronometer for each mouse. The recovery of the righting reflex was confirmed by the ability of the mice to re-right 3 times within 1 min. The drugs were freshly prepared before use, and the total injection volume (13 mL/kg) was retained constant.

### Open Field Test

Male BALB/cAnNRj mice (9–11 weeks of age) were habituated to the testing room in their home cages for 1 h before treatment. Animals were randomly assigned to three groups where each mouse was injected *i.p.* with either 10 or 20 mg/kg humulone or vehicle (control) 45 min before the test. Each mouse was then placed individually into the center of a brightly illuminated white Plexiglass arena (50 × 50 × 38 cm) and recorded continuously for 15 min with Ethovision version 13 tracking system (Noldus, Wageningen, The Netherlands). The parameters of locomotor activity such as distance and velocity were quantified and analyzed for the whole and subsections of the arena. All arena surfaces were thoroughly cleaned with 70% ethanol and dried between each tested subject.

### Statistical Analysis

Data presentation, curve fitting and statistical analysis were performed using GraphPad Prism 8 software package (GraphPad, San Diego, CA, United States). Concentration series data were fitted with a sigmoidal dose-response (variable Hill Slope) equation using non-linear least squares regression to estimate the IC_50_ (half-maximal inhibitory concentration) values for non-competitive radioligand binding. Statistical comparisons were made with One-way ANOVA followed by relevant (Dunnett’s or Tukey’s) *post hoc* tests for multiple comparisons. To evaluate the statistical difference between only two groups, Student’s *t*-test was selected. All data were expressed as Means ± SEM and *p*-values of less than 0.05 denoted a statistically significant difference.

## Results

### Modulation of GABA-Induced Currents by Humulone in Recombinant GABA_A_ Receptors

Given the well-established role of α1 subunit in GABA_A_ receptor-mediated sedative effects (Rudolph et al., 1999; McKernan et al., 2000), the functional interaction of humulone with GABA_A_ receptor complex was confirmed electrophysiologically in recombinant α1β3γ2 GABA_A_ receptors expressed in HEK293 cells. Using whole-cell patch-clamp recordings, we assigned a submaximal concentration of GABA at 1 μM to highlight the potentiating effect of humulone. Transfected HEK293 cells were voltage-clamped at −60 mV, and receptors were activated upon 1 min application of GABA (1 μM) in the presence or absence of humulone (10 μM). GABA-induced currents were significantly potentiated by humulone as illustrated in representative current traces (Figure 1B). Peak current amplitudes were normalized to that induced by the presence of 1 μM GABA alone (Figure 1C), where the calculated mean of humulone potentiation was 158 ± 41% (*p* < 0.01, *n* = 8 cells).

### Humulone-Ethanol Interaction in [^3^H]EBOB Binding With Native GABA_A_ Receptors

**FIGURE 2:**
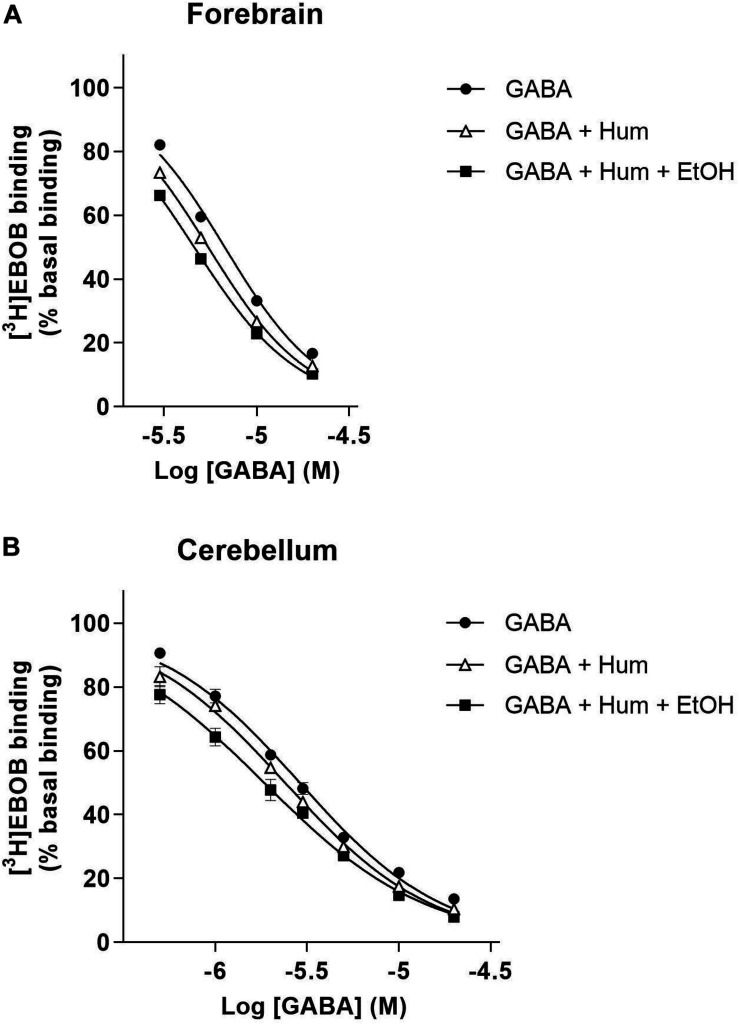
GABA concentration curve of [^3^H]EBOB displacement in forebrain **(A)** and cerebellar **(B)** membranes with humulone (1 μM) in the presence or absence of EtOH (30 mM). All values represent the mean ± SEM, *n* = 3, measured in triplicates.

We examined ethanol enhancement of humulone’s modulatory effects on [^3^H]EBOB binding to forebrain and cerebellar membranes. GABA concentration series indicated that in the presence of low humulone concentration (1 μM), further inclusion of ethanol (30 mM) produces a leftward shift of GABA-induced [^3^H]EBOB displacement curve (Figure 2). Analysis with One-way ANOVA followed by Tukey’s *post hoc* test showed that ethanol decreased the IC_50_ of GABA-induced [^3^H]EBOB displacement from 5.48 ± 0.09 μM to 4.60 ± 0.08 μM in the forebrain (*p* < 0.01) and to a higher extent from 2.40 ± 0.06 μM to 1.80 ± 0.07 μM in the cerebellum (*p* < 0.01) (Table 1).

**TABLE 1:** Effects of GABA on [^3^H]EBOB binding in rat forebrain and cerebellar membranes with humulone in the presence or absence of ethanol (EtOH).

Membrane	IC_50_ (μM)		
	GABA	GABA + 1 μM Humulone	GABA + 1 μM Humulone + 30 mM EtOH
Forebrain	6.73 ± 0.18	5.48 ± 0.09**	4.60 ± 0.08
Cerebellum	2.89 ± 0.09	2.40 ± 0.06**	1.80 ± 0.07

The IC_50_ values represent mean ± SEM (n = 3, measured in triplicates). **p < 0.01, significantly different from the corresponding IC_50_ of GABA alone and GABA+Humulone+EtOH (One-way ANOVA followed by Tukey’s post hoc test).

**FIGURE 3:**
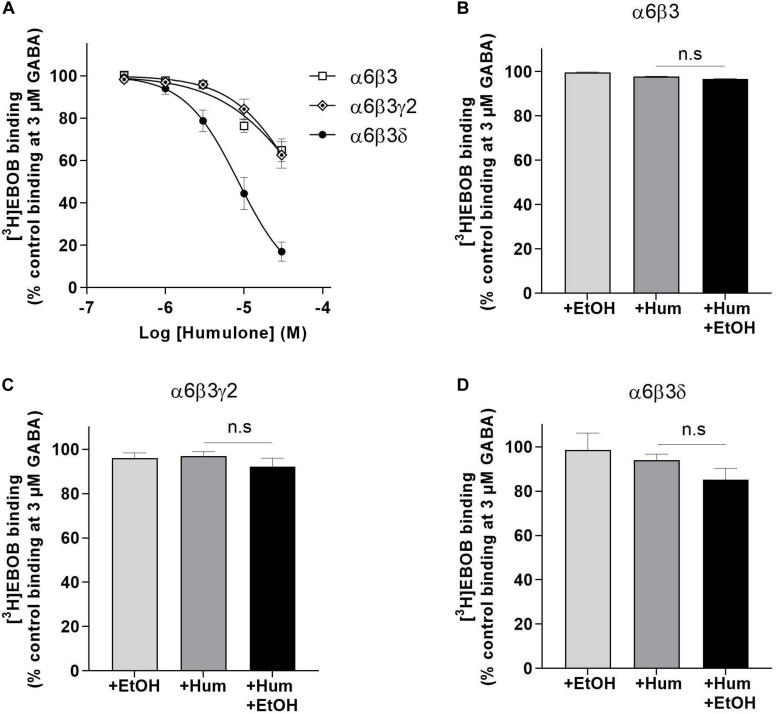
Modulation of GABA-induced [^3^H]EBOB displacement in recombinant α6β3, α6β3γ2 and α6β3δ GABA_A_ receptors expressed in HEK293 cells. **(A)** Displacement curves of [^3^H]EBOB (1 nM) binding as % control with 5 concentrations of humulone in the presence of GABA (3 μM). **(B–D)** GABA (3 μM)-induced [^3^H]EBOB (1 nM) binding as % control with humulone (1 μM) in the presence or absence of EtOH (30 mM). Control is the maximal [^3^H]EBOB binding in the presence of 3 μM GABA alone. All values represent the mean ± SEM, *n* = 3–6, measured in triplicates; n.s for the non-significance of difference from the corresponding control value (One-way ANOVA followed by Tukey’s *post hoc* test).

We assessed the influence of γ and δ subunits on humulone’s modulatory effects using [^3^H]EBOB binding in recombinant α6β3γ2, α6β3δ, and α6β3 receptors expressed in HEK293 cells. As shown in Figure 3A, humulone potentiated GABA-induced [^3^H]EBOB displacement dose-dependently in all tested receptor subtypes. This potentiation was evident with humulone at concentrations down to 1 μM in α6β3δ receptors, while such effect in α6β3, α6β3γ2 receptors was only observed with humulone at 10 μM concentration and above. The IC_50_ values of humulone in the presence of GABA (3 μM) indicated higher inhibition potency in α6β3δ receptor subtype (8.45 ± 0.92 μM) compared to α6β3γ2 (49.03 ± 10.11 μM) and α6β3 (56.85 ± 12.79 μM) (*p* < 0.01), with no statistically significant difference between α6β3γ2 and α6β3 receptor subtypes (One-way ANOVA followed by Tukey’s *post hoc* test).

We then verified whether humulone modulation is sensitive to low ethanol dose (30 mM) in recombinant α6β3, α6β3γ2, and α6β3δ receptors as observed in forebrain and cerebellum. Humulone (1 μM) effect on [^3^H]EBOB displacement induced by GABA (3 μM) was absent in α6β3 and α6β3γ2 receptor subtypes and co-incubation with ethanol did not alter this state (unpaired *t*-test) (Figures 3B,C). Similarly, ethanol alone showed no effect on GABA-induced [^3^H]EBOB displacement and did not significantly enhance humulone modulation in α6β3δ receptor subtype (One-way ANOVA followed by Tukey’s *post hoc* test) (Figure 3D).

### The Additive Modulation of [^3^H]EBOB Binding by Humulone and Other Hops Compounds With Native GABA_A_ Receptors

**FIGURE 4:**
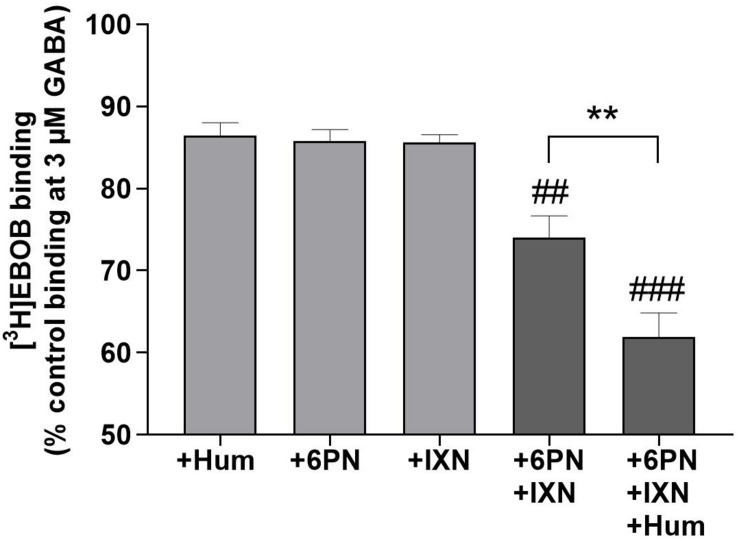
Additive potentiation of GABA-induced [^3^H]EBOB displacement in rat forebrain with humulone combined with 6PN and IXN at 1 μM. Control is the maximal [^3^H]EBOB binding in the presence of 3 μM GABA alone. Each vertical bar represents the mean ± SEM, *n* = 3, measured in triplicates.^###^*p* < 0.001, ^##^*p* < 0.01 for the significance of difference from the corresponding individual compounds, **p < 0.01 for the significance of difference between 6PN+IXN+Hum and 6PN+IXN (One-way ANOVA followed by Tukey’s *post hoc* test).

We evaluated the interactions between humulone and other reported hops compounds active at GABA_A_ receptors (Benkherouf et al., 2020) to modulate [^3^H]EBOB binding to GABA_A_ receptors at low micromolar concentrations. In the presence of 3 μM GABA, combination of 6-prenylnaringenin (6PN) and isoxanthohumol (IXN) at 1 μM led to an additive potentiation of GABA-induced [^3^H]EBOB displacement (*p* < 0.01) in rat forebrain. Further co-incubation with humulone significantly increased this potentiation observed with 6PN+IXN combination (*p* < 0.01) (Figure 4). These additive effects corresponded with the sum of [^3^H]EBOB displacement by each compound individually as shown in Table 2.

**TABLE 2:** [^3^H]EBOB displacement normalized to the maximum radioligand binding in the presence of 3 μM GABA alone.

Compound (1 μM)	[^3^H]EBOB displacement (%)
Hum	13.5 ± 1.5
6PN	14.2 ± 1.4
IXN	14.4 ± 0.9
6PN+IXN	25.9 ± 2.6
6PN+IXN+Hum	38.1 ± 2.9

**Values represent mean ± SEM (n = 3, measured in triplicates).**

### Sleep-Enhancing Actions of Humulone in Pentobarbital- and Ethanol-Induced Sleep in Mice

**FIGURE 5:**
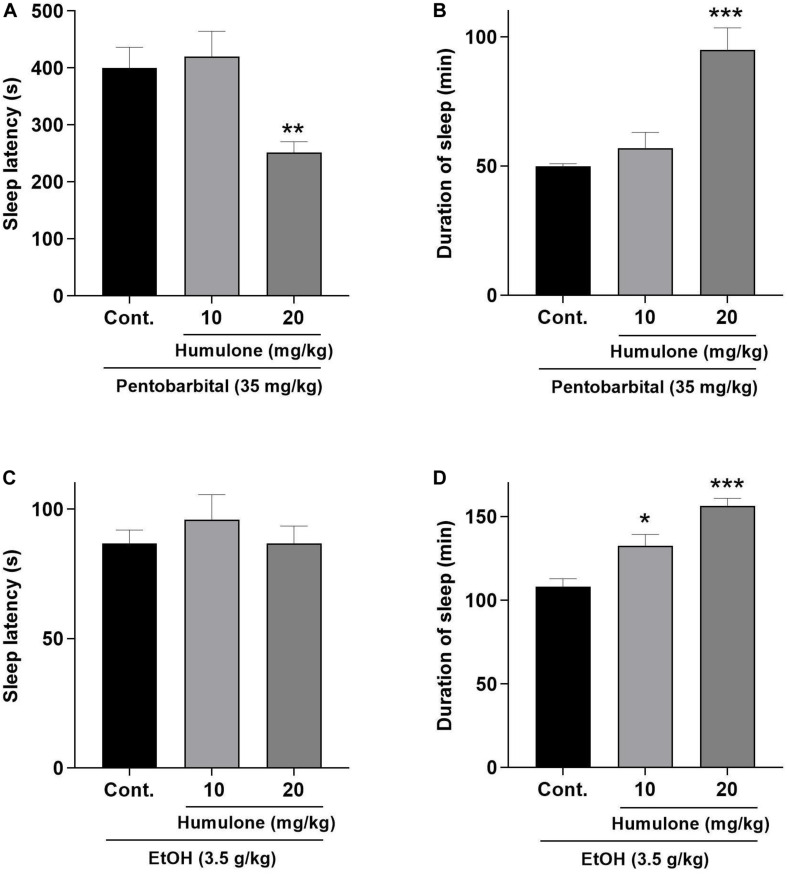
Effects of humulone on sleep latency, in seconds (s) and duration of sleep, in minutes (min) induced by pentobarbital sodium (35 mg/kg, *i.p*.) **(A,B)** and ethanol (3.5 g/kg, *i.p*.) **(C,D)** in mice. Each vertical bar represents the mean ± SEM, *n* = 6–7 mice/group. ***p < 0.001, **p < 0.01, *p < 0.05 for the significance of difference from the corresponding vehicle control group (One-way ANOVA followed by Dunnett’s *post hoc* test).

The effects of humulone pre-treatment on the latency and duration of sleep induced by sodium pentobarbital (35 mg/kg, *i.p*.) and ethanol (3.5 g/kg) in mice are presented in Figure 5. Humulone at 20 mg/kg dose significantly decreased the latency (*p* < 0.01) and prolonged the duration of sleep (*p* < 0.001) induced by pentobarbital compared to the control group. These effects were not observed at a lower humulone dose of 10 mg/kg. On the other hand, despite humulone showed no effect on the onset of sleep induced by ethanol, it significantly increased sleep duration dose-dependently at both 10 mg/kg (*p* < 0.05) and 20 mg/kg (*p* < 0.001) compared to control.

### The Effects of Humulone on Spontaneous Locomotor Behavior in Mice

**FIGURE 6:**
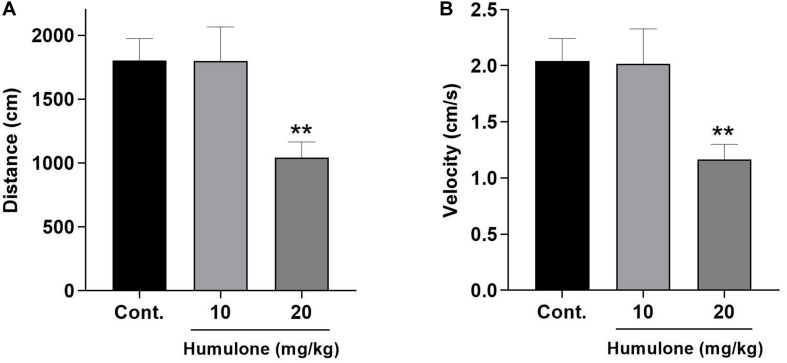
Effects of acute humulone exposure (10 and 20 mg/kg) on the locomotor activity of mice in open field test. Distance traveled **(A)** and velocity **(B)** were recorded during 15 min observation period, 45 min after intraperitoneal administration of humulone or vehicle. Each vertical bar represents the mean ± SEM, *n* = 7–11 mice/group. **p < 0.01 for the significance of difference from the corresponding vehicle control group (One-way ANOVA followed by Dunnett’s *post hoc* test).

Open field test was carried out to evaluate the spontaneous locomotor activity in humulone-treated mice as an index for sedation (Figure 6). Two doses of humulone were assessed (10 and 20 mg/kg, *i.p*.) for their effects, 45 min after administration over a 15 min observation period. One-way ANOVA followed by Dunnett’s *post hoc* test revealed a significant effect of the higher dose of humulone on the tested locomotor parameters. Mice treated with humulone at 20 mg/kg traveled a shorter total distance (*p* < 0.01) with lower average velocity (*p* < 0.01) compared to the vehicle control group. However, the significant difference in these two parameters was not observed with humulone at 10 mg/kg dose vs. control.

## Discussion

In the present study, we demonstrated electrophysiologically for the first time humulone’s positive allosteric modulation of GABA_A_ receptor function, displayed its sedative activity and enhancement of pentobarbital and ethanol hypnotic effects. This activity can be presumably enhanced by ethanol and GABA_A_R-active hops modulators as suggested by our [^3^H]EBOB binding results in brain membranes.

We reported earlier that humulone potentiates GABA-induced [^3^H]EBOB displacement in native GABA_A_ receptors at low micromolar concentrations (Benkherouf et al., 2020). Hence, it was essential to demonstrate humulone’s ability in modulating of GABA_A_ receptor function using electrophysiological measurements. The results indicate a significant potentiation of non-saturating GABA-induced currents by humulone (10 μM) in the highly abundant α1β3γ2 receptor subtype (Wisden et al., 1992; Fritschy and Mohler, 1995), which contains α1 subunit that plays a key role in GABA_A_ receptor-mediated sedative effects (Rudolph et al., 1999; McKernan et al., 2000). This confirms humulone’s mode of action as a positive allosteric modulator of GABA_A_ receptors, in accordance with the published reports on the enhancement of GABA-induced currents by hops extracts (Aoshima et al., 2006; Sahin et al., 2016). The correlation between electrophysiology and [^3^H]EBOB binding results supports the latter’s advantage in assessing drug enhancement of GABA_A_ receptor function as, similarly, confirmed with [^35^S]TBPS (Im and Blakeman, 1991; Sieghart, 1995; Atucha et al., 2009).

Since humulone dietary intake occurs mainly through beer consumption, this prompted further exploration into humulone-ethanol interaction in GABA_A_ receptors using [^3^H]EBOB binding assay, given the evident potentiating effect of humulone on the duration of sleep induced by ethanol. Despite ethanol solely neither displaces nor modulates [^3^H]EBOB binding at low millimolar concentrations (<30 mM) (Supplementary Figure S2; Höld et al., 2000; Zhao et al., 2014), it was found to enhance humulone modulation of [^3^H]EBOB binding in both forebrain and cerebellar membranes. However, the extent of such humulone non-competitive synergy with ethanol was 11.2% higher in cerebellum compared to forebrain (Table 1). The cerebellum is a major center for motor coordination and control, where it plays an important role in the acute and chronic effects of alcohol (Eidelberg et al., 1971; Seiger et al., 1983; Engblom et al., 1991; Luo, 2015; Valenzuela and Jotty, 2015). Excessive alcohol consumption is associated with cerebellar ataxia and postural instability, which can persist with long-term exposure as a consequence of cerebellar volume reduction and vermis damage (Sullivan et al., 2002; Sullivan and Pfefferbaum, 2005; Jaatinen and Rintala, 2008). Moreover, α6βxδ GABA_A_ receptors are highly expressed in cerebellum and found to display high sensitivity to ambient GABA and ethanol (Wisden et al., 1992; Quirk et al., 1995; Saxena and Macdonald, 1996; Pirker et al., 2000; Sundstrom-Poromaa et al., 2002; Wallner et al., 2003; Hanchar et al., 2005). However, α6βxδ low millimolar sensitivity to ethanol remains controversial as several groups reported opposing findings (Borghese et al., 2006; Yamashita et al., 2006; Korpi et al., 2007; Baur et al., 2009). Nevertheless, since α6-containing GABA_A_ receptors are expressed almost exclusively in cerebellar granule cells (Jechlinger et al., 1998; Nusser et al., 1998; Pirker et al., 2000; Pöltl et al., 2003), we assessed their involvement in humulone-ethanol interactions observed in cerebellar membranes.

Based on our results, humulone was able to modulate [^3^H]EBOB binding in recombinant α6β3δ receptors in the absence of ethanol with exceptional higher potency compared to α6β3γ2 and α6β3. While the incorporation of γ2 subunit into α6β3 receptor subtype did not significantly alter humulone’s modulatory behavior (Figure 3A), δ subunit led to an increase in humulone potency by 5.8 and 6.7 fold compared to α6β3γ2 and α6β3 receptors, respectively. These findings indicate a key role of δ subunit in the execution of humulone’s low micromolar effects on extrasynaptic GABA_A_ receptors, an important target for anesthetics (propofol), sleep-promoting drugs (gaboxadol), neurosteroids (allopregnanolone), and alcohol (Brickley and Mody, 2012; Houston et al., 2012). Nevertheless, humulone’s modulation of α6-containing GABA_A_ receptors suggests behavioral implications in motor coordination which deserve further investigation based on the role of α6 subunit in mediating alcohol and benzodiazepine-induced ataxia (Korpi et al., 1999; Hanchar et al., 2005).

Given the demonstrated low potency of ethanol in [^3^H]EBOB displacement (IC_50_ = 370 ± 4 mM) (Höld et al., 2000; Zhao et al., 2014), our results show that GABA-induced [^3^H]EBOB binding was unaffected by the presence of ethanol at 30 mM in α6β3, α6β3γ2, and α6β3δ receptor subtypes. Moreover, in contrast to our observations in this study with forebrain and cerebellar membranes, the co-incubation of ethanol (30 mM) with humulone (1 μM) did not enhance humulone-mediated [^3^H]EBOB displacement in α6β3δ receptor subtype as well as α6β3γ2 and α6β3. An electrophysiological study showed a comparable observation where low ethanol dose did not influence the chloride channel kinetics in recombinant α1β2γ2 GABA_A_ receptors expressed in HEK293 cells (Akk and Steinbach, 2003). In the same study, co-application of ethanol with the neurosteroid 3α-hydroxy-5α-androstane-17β-carbonitrile (ACN) increased the open probability of the channel, but it did not influence receptor affinity or the extent of channel opening, which are determinant states for [^3^H]EBOB displacement and modulation of GABA_A_ receptor function. Furthermore, ethanol sensitivity at low millimolar doses in recombinant δ-containing GABA_A_ receptors remains debatable as previously reviewed (Förstera et al., 2016). Ethanol was reported to bind competitively to [^3^H]Ro 15–4513 binding site in α4/6βxδ receptors (Hanchar et al., 2006; Wallner et al., 2006b; Santhakumar et al., 2007), but neither [^3^H]Ro 15–4513 binding nor its displacement by ethanol have been detected in α4/6βxδ receptors by other groups (Borghese and Harris, 2007; Korpi et al., 2007). The complexity of ethanol actions on native GABA_A_ receptors and the involvement of presynaptic mechanisms and protein kinase phosphorylation (PKC and PKA) in modulating ethanol sensitivity renders a precise simulation in expression systems challenging (Harris et al., 1995; Freund and Palmer, 1997; Weiner et al., 1997; Aguayo et al., 2002; Carta et al., 2004). Hence, despite the relatively high potency of humulone on α6β3δ GABA_A_ receptor subtype, its role in the cerebellar ethanol-humulone synergy is yet inconclusive. Nevertheless. it was evident that humulone exhibits a very weak displacement in [^3^H]Ro 15–4513 binding to native αβγ2 GABA_A_ receptors and its modulatory effect on [^3^H]EBOB binding is insensitive to flumazenil antagonism (Benkherouf et al., 2020). Hence, our observed non-competitive synergistic effect with ethanol in brain membranes and humulone’s differential potency in recombinant receptors further confirms that the humulone modulatory site is not the classical benzodiazepine binding site in the α+γ2- interface.

In this paper, we found that the modulatory activity of IXN, the most abundant flavonoid in beer, can be additively enhanced by another flavonoid, 6PN, which was detected in beer as well (Stevens et al., 1999). Interestingly, we noted that humulone interacts with these modulators leading to further potentiation that corresponds to the sum of [^3^H]EBOB displacement by each compound individually. These findings correlate with the earlier proposed differences between 6PN and IXN in molecular docking at GABA_A_ receptor α1β2γ2 isoform where a higher binding free energy was predicted for 6PN at both α1+β2- and α1+γ2- interfaces (Benkherouf et al., 2020). Consistently, 6PN was remarkably more efficient than IXN and humulone in displacing [^3^H]Ro 15–4513 binding to the classical benzodiazepine binding site in GABA_A_ receptors suggesting a favorable binding site for 6PN at α1+γ2- interface (Benkherouf et al., 2019, 2020). However, it is unlikely that 6PN additive modulation with IXN and humulone occurs via α1+γ2- interface due to 6PN insensitivity to flumazenil antagonism and hence the lack of involvement of the classical benzodiazepine site in its allosteric activity. The modulatory enhancements between 6PN and IXN may still occur via α1+β2- interface since molecular docking revealed additional receptor residues interacting with 6PN but not with IXN: Lys156, Gln204 and Ser205 (Benkherouf et al., 2020). The positive modulatory interactions between hops prenylflavonoids and humulone was similarly noted between some flavonoids and other positive modulators acting on GABA_A_ receptors. For instance, apigenin (chamomile) and (–)-epigallocatechin gallate (green tea) were found to potentiate diazepam modulation in recombinant α1β1γ2 receptors (Campbell et al., 2004). Furthermore, flavonoid positive modulators from *Valeriana* species such as 6-methylapigenin and linarin act synergistically with hesperidin and valerenic acid, respectively, displaying enhancements in sleep induction *in vivo* (Marder et al., 2003; Fernández et al., 2004). Supported by the fact that positive allosteric modulators binding to different binding sites may exhibit additive effects (Visser et al., 2003; McMahon and France, 2005; Paronis, 2006), we propose that hops neuroactivity may involve more than one compound leading to enhanced potentiation of GABA_A_ receptors function.

Humulone alpha acid appears to play a significant role in hops sedative and hypnotic behavior. It was demonstrated earlier that alpha acid extract increases pentobarbital-induced sleep duration in rats with no alteration in sleep onset (Zanoli et al., 2005). On the other hand, our results with mice showed that humulone increased sleep duration and decreased sleep onset as well. Consistently, humulone component in alpha acid extract at 21 mg/kg was shown to increase the duration of sleep induced by ketamine in mice with no additional effect noted at 42 mg/kg (Schiller et al., 2006). These doses were below humulone’s LD_50_ values documented for rodents: 1,500 mg/kg/b.w. *p.o.*; 600 mg/kg/b.w. *i.m.* (rats) (Bejeuhr, 1993). We further confirmed humulone’s hypnotic effects with ethanol to rule out the possibility of pharmacokinetic interactions with humulone leading to enhancement in pentobarbital-induced sleep. Interestingly, humulone at a lower dose of 10 mg/kg increased the duration of sleep induced by ethanol but not by pentobarbital. The Human Equivalent Dose (HED) based on body surface area for 10 mg/kg in mice is 0.81 mg/kg (Nair and Jacob, 2016). Noting that humulone solubility is 14 mg/L in beer and was detected up to 28 mg/L (Fritsch and Shellhammer, 2007), the dose of 10 mg/kg is comparable to a 60 kg human consuming 2 L of hopped beer with a humulone concentration of 24.2 mg/L (Hahn et al., 2018). Despite humulone did not alter the onset of sleep induced by ethanol, the evident increase in the duration of sleep could be attributed to the synergistic interaction with ethanol on native GABA_A_ receptors as discussed above.

As an index for sedation, an early study reported that 100 and 200 mg/kg of alpha acid extract containing 36% of humulone decreased locomotor activity in open field test and showed no indications for anxiolytic activity in elevated plus maze test (Schiller et al., 2006). Correspondingly, our results displayed a similar effect on locomotion and at a lower humulone dose of 20 mg/kg. This is not a likely alteration in anxiety behavior since no differences in open field were noted in the time spent in periphery and center between all tested mice groups (Supplementary Figure S3). On the other hand, in rats, the decrease in locomotion was not observed with alpha acid extract at 10 and 20 mg/kg (Zanoli et al., 2005). The presence of several weak modulators that potentially compete for the same binding site as humulone may reduce the potency of alpha acids. This is because alpha acids contain cohumulone and adhumulone, which are structurally very similar to humulone and display weak modulatory effects on GABA_A_ receptors (Benkherouf et al., 2020).

## Conclusion

In conclusion, humulone, a major compound in hops, exhibits sedative/hypnotic effects and acts as a positive allosteric modulator of GABA_A_ receptors. This supports humulone’s substantial role in hops sleep-promoting activity and brings further insight into the probable mode of action for this behavior. Hops flavonoids such as IXN and 6PN may potentiate humulone effects via additive mechanisms on GABA_A_ receptors. Moreover, the displayed humulone non-competitive synergy with ethanol in GABA_A_ receptors may contribute to further enhancement in alcohol intoxication with high-hopped beer. Hence, the implication of this on alcohol drinking patterns and reward in humans needs further investigation. Nevertheless, the identification of neuroactive compounds from hops and understanding their interactions advance the development of safe and efficacious remedies for insomnia and sleep disturbances.

## Data Availability Statement

The original contributions presented in the study are included in the article/Supplementary Material, further inquiries can be directed to the corresponding author.

## Ethics Statement

The animal study was reviewed and approved by the National Animal Experiment Board in Finland.

## Author Contributions

AB: investigation, methodology, formal analysis, data curation, visualization, project management, writing—original draft, writing—review and editing. KE: investigation, methodology, writing—review and editing. SS: investigation, methodology, writing—review and editing. MU-O: conceptualization, resources, methodology, formal analysis, validation, funding acquisition, supervision, writing—review and editing. All authors have participated sufficiently in the work to take public responsibility for the content, including participation in the concept, design, analysis, writing, or revision of the manuscript.

## Conflict of Interest

The authors declare that the research was conducted in the absence of any commercial or financial relationships that could be construed as a potential conflict of interest.
