# Supplementary material for: Humulone Modulation of GABAA Receptors and Its Role in Hops Sleep-Promoting Activity
Source: Front Neurosci. 2020 Oct 14;14:594708. doi: 10.3389/fnins.2020.594708 (PMC7591795; doi:10.3389/fnins.2020.594708)

Supplementary Material

**Figure S1.|** [^3^H]Ro 15-4513 binding to recombinant α6β3 and α6β3γ2 receptors expressed in HEK293 cells. Triplicate samples were incubated on ice at 4 °C with shaking for 1 h in assay buffer (10 mM Tris-HCl, pH 7.4) with [^3^H]Ro 15-4513 (2 nM) in a total volume of 300 μl. Non-specific binding was determined with flumazenil (10 μM). The protein concentrations were determined with NanoDrop™ One Microvolume UV-Vis Spectrophotometer (Thermo Fisher Scientific, Waltham, MA, USA) according to manufacturer’s instructions. ****p < 0.0001 for the significance of difference from [^3^H]Ro 15-4513 specific binding to α6β (unpaired t-test).

**
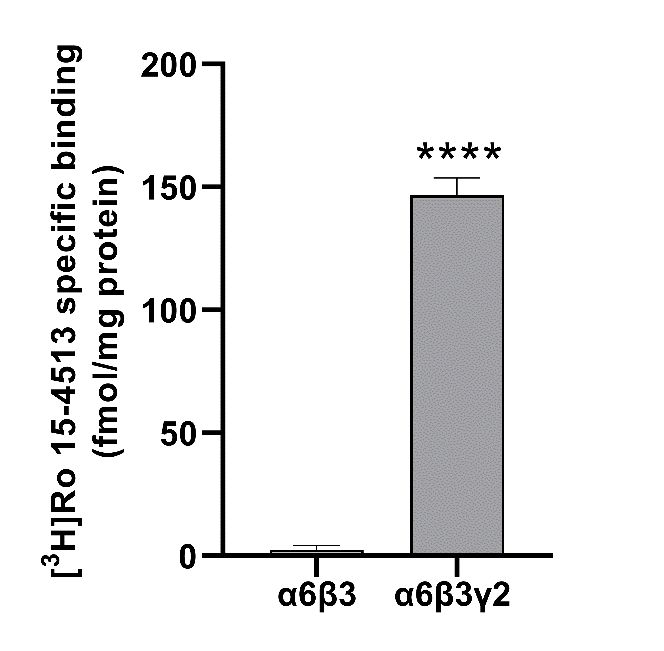
**

**Figure S2.|** Displacement curves of EtOH-free [^3^H]EBOB (1 nM) binding to rat cerebellar membranes with incubating different concentrations (0.3-30 mM) of ethanol in the presence or absence of 10 nM/1 µM GABA. All values represent the mean ± SEM, n = 3-4, measured in triplicates.


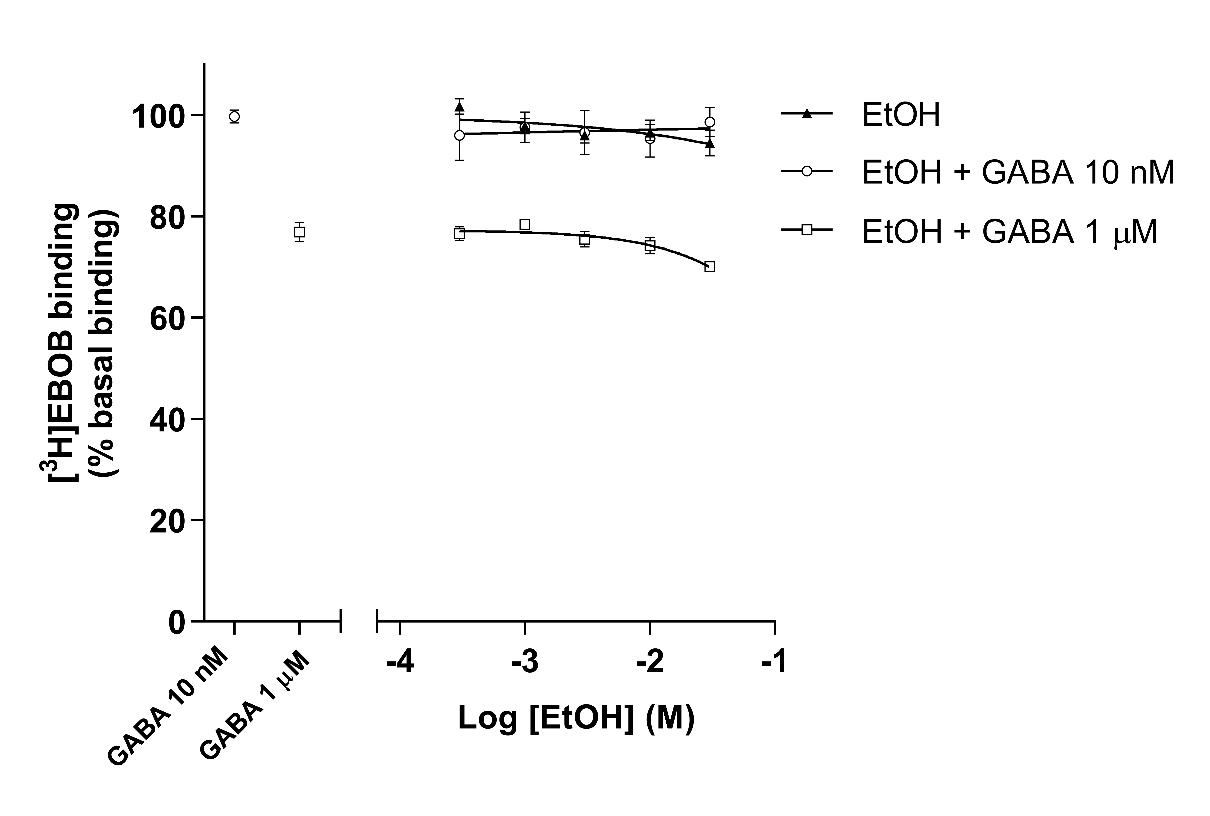


**Figure S3.|** | Effects of acute humulone exposure (10 and 20 mg/kg) on the locomotor activity of mice in open field test. Time spent in periphery **(A)** and time spent in center **(B)** were recorded during 15 min observation period, 45 min after intraperitoneal administration of humulone or vehicle. Each vertical bar represents the mean ± SEM, n = 7-11 mice/group. n.s for the non-significance of difference between all values (One-way ANOVA followed by Tukey’s *post hoc* test).


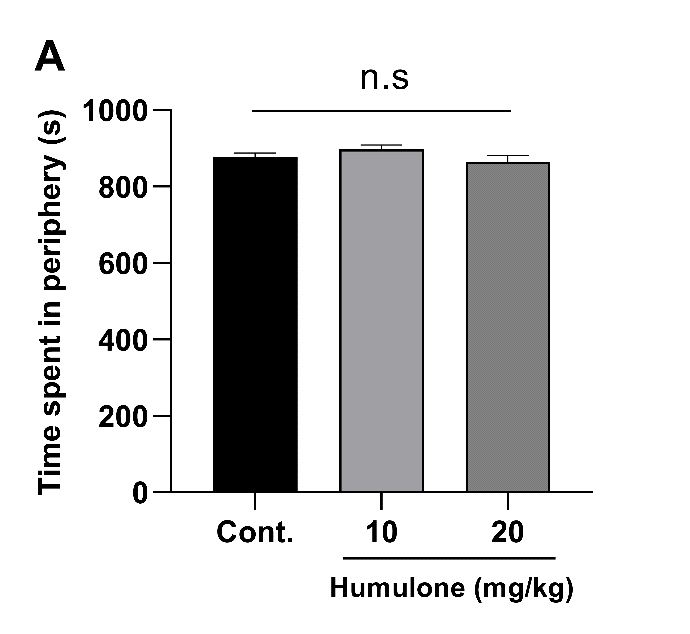

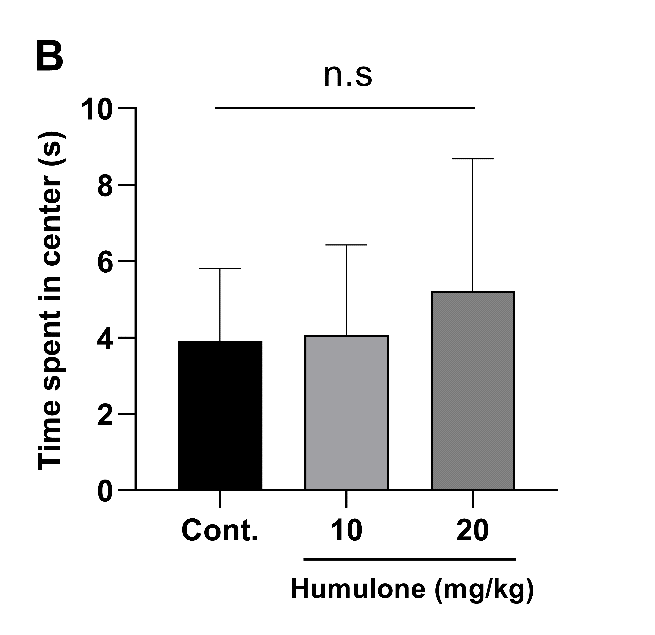

Supplement: Supplementary file 1 [file Data_Sheet_1.docx]
